# 3-Chloro-*N*-cyclo­hexyl­benzamide

**DOI:** 10.1107/S1600536809017012

**Published:** 2009-05-14

**Authors:** M. Khawar Rauf, Michael Bolte, Amin Badshah

**Affiliations:** a Department of Chemistry, Quaid-i-Azam University, Islamabad 45320, Pakistan; b Institut für Anorganische Chemie, J. W. Goethe-Universität Frankfurt, Max-von-Laue-Strasse 7, 60438 Frankfurt/Main, Germany; c Department of Chemistry, Islamia University of Bahawalpur, Pakistan

## Abstract

In the title mol­ecule, C_13_H_16_ClNO, the mean plane of the atoms in the –CONH– group forms a dihedral angle of 42.0 (4)° with the benzene ring plane. In the crystal structure, mol­ecules are linked by inter­molecular N—H⋯O hydrogen bonds, generating *C*(4) chains along [100].

## Related literature

For bond-length data, see: Allen (2002 ▶). For related structures, see: Garden *et al.* (2005 ▶); Wardell *et al.* (2005 ▶). For hydrogen-bond motifs, see: Bernstein *et al.* (1995 ▶).
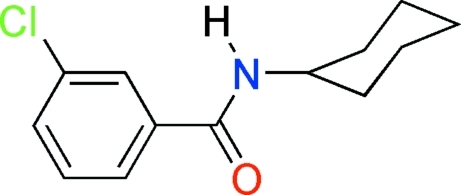


## Experimental

### 

#### Crystal data

C_13_H_16_ClNO
*M* *_r_* = 237.72
Orthorhombic, 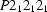

*a* = 8.4963 (6) Å
*b* = 11.4891 (7) Å
*c* = 12.5318 (11) Å
*V* = 1223.29 (16) Å^3^
*Z* = 4
Mo *K*α radiation
μ = 0.29 mm^−1^
*T* = 173 K
0.38 × 0.22 × 0.22 mm

#### Data collection

Stoe IPDS II two-circle diffractometer
Absorption correction: multi-scan [*MULABS* (Spek, 2003 ▶; Blessing, 1995 ▶)] *T*_min_ = 0.898, *T*_max_ = 0.939
6758 measured reflections
2737 independent reflections
2429 reflections with *I* > 2σ(*I*)
*R*_int_ = 0.035

#### Refinement

*R*[*F* ^2^ > 2σ(*F* ^2^)] = 0.030
*wR*(*F* ^2^) = 0.069
*S* = 0.98
2737 reflections
150 parameters
H atoms treated by a mixture of independent and constrained refinement
Δρ_max_ = 0.18 e Å^−3^
Δρ_min_ = −0.17 e Å^−3^
Absolute structure: Flack (1983 ▶), 1128 Friedel pairs
Flack parameter: 0.03 (5)

### 

Data collection: *X-AREA* (Stoe & Cie, 2001 ▶); cell refinement: *X-AREA*; data reduction: *X-AREA*; program(s) used to solve structure: *SHELXS97* (Sheldrick, 2008 ▶); program(s) used to refine structure: *SHELXL97* (Sheldrick, 2008 ▶); molecular graphics: *SHELXTL-Plus* (Sheldrick, 2008 ▶); software used to prepare material for publication: *SHELXL97*.

## Supplementary Material

Crystal structure: contains datablocks I, global. DOI: 10.1107/S1600536809017012/lh2817sup1.cif

Structure factors: contains datablocks I. DOI: 10.1107/S1600536809017012/lh2817Isup2.hkl

Additional supplementary materials:  crystallographic information; 3D view; checkCIF report

## Figures and Tables

**Table 1:** Hydrogen-bond geometry (Å, °)

*D*—H⋯*A*	*D*—H	H⋯*A*	*D*⋯*A*	*D*—H⋯*A*
N1—H1⋯O1^i^	0.868 (18)	2.052 (18)	2.9161 (15)	173.3 (16)

Symmetry code: (i) 
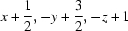
.

**Fig. 1.**
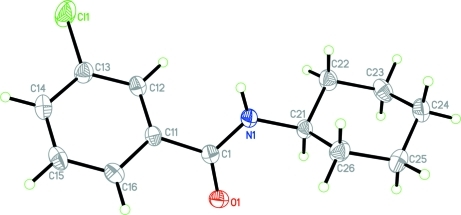
Molecular structure of (I) showing atom numbering scheme. Displacement ellipsoids are drawn at the 50% probability level.

**Fig. 2.**
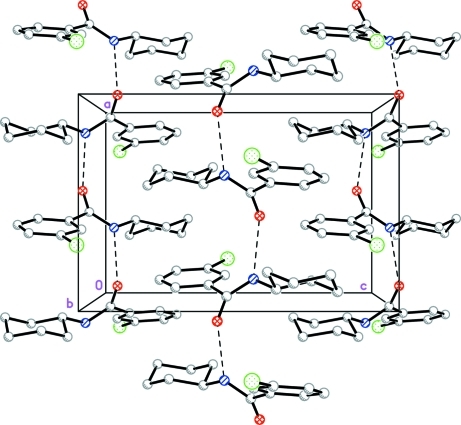
Part of the crystal structure of (I) viewed onto the ac plane. H atoms are omitted for clarity. Dashed lines are drawn between the non-hydrogen donor and acceptor atoms of hydrogen bonds.

## supplementary crystallographic information

### Comment

We report herein the structure of the title compound, (I) (Fig. 1), which was separated from an impure sample of thiourea by column chromatography as a byproduct, as part of our ongoing studies related to *N*,*N*'-disubstituted thioureas and heterocyclic compounds. In this class of componds, N—H···O, C—H···O and N—H···N hydrogen bonds, and weak π–π stacking interactions are the only direction-specific intermolecular interactions (Garden *et al.*, 2005; Wardell *et al.*, 2005). In the crystal structure, molecules form intermolecular N—H···O hydrogen bonds to generate *C*(4) chains (Bernstein *et al.*,  1995) along [100] (Fig. 2). The molecules of (I) are not planar, as evidenced by the torsion angles (C21—N1—C1—O1, 2.9 (02) and C21—N1—C1—C11, -174.88 (11)°) associated with –CONH– moiety, and the amide group adopts the usual *trans* conformation; the bond lengths (Allen, 2002) and inter-bond angles present no unusual values.

### Experimental

Freshly prepared and steam distillated 3-chlorobenzoyl isothiocyanate (1.97 g, 10 mmol) was stirred in acetone (30 ml) for 20 min. Neat cyclohexylamin (1.0 g, 10 mmol) was then added and the resulting mixture was stirred for 1 h. The reaction mixture was then poured into 300 ml (approx.) acidified (pH 4) water and stirred well. The solid product was separated and washed with deionized water. One of the fraction obtained during the column chromatography of the target thiourea was recrystallized from methanol/1,1-dichloromethane (1:10 *v*/*v*) to give fine crystals of (I), with an overall fractional yield of 15%.

### Refinement

H atoms bonded to C were included in calculated positions and refined as riding on their parent C atom with C—H ranging from 0.93 Å to 1.0 Å and *U*_iso_(H) = 1.2*U*_eq_(C). The H atom bonded to N was freely refined.

### Crystal data

**Table:**

C_13_H_16_ClNO	*F*(000) = 504
*M**_r_* = 237.72	*D*_x_ = 1.291 Mg m^−^^3^
Orthorhombic, *P*2_1_2_1_2_1_	Mo *K*α radiation, λ = 0.71073 Å
Hall symbol: P 2ac 2ab	Cell parameters from 6652 reflections
*a* = 8.4963 (6) Å	θ = 3.4–27.7°
*b* = 11.4891 (7) Å	µ = 0.29 mm^−^^1^
*c* = 12.5318 (11) Å	*T* = 173 K
*V* = 1223.29 (16) Å^3^	Block, colourless
*Z* = 4	0.38 × 0.22 × 0.22 mm

### Data collection

**Table:**

Stoe IPDS II two-circle diffractometer	2737 independent reflections
Radiation source: fine-focus sealed tube	2429 reflections with *I* > 2σ(*I*)
graphite	*R*_int_ = 0.035
ω scans	θ_max_ = 27.5°, θ_min_ = 3.4°
Absorption correction: multi-scan [*MULABS* (Spek, 2003; Blessing, 1995)]	*h* = −11→11
*T*_min_ = 0.898, *T*_max_ = 0.939	*k* = −14→11
6758 measured reflections	*l* = −16→13

### Refinement

**Table:**

Refinement on *F*^2^	Hydrogen site location: inferred from neighbouring sites
Least-squares matrix: full	H atoms treated by a mixture of independent and constrained refinement
*R*[*F*^2^ > 2σ(*F*^2^)] = 0.030	*w* = 1/[σ^2^(*F*_o_^2^) + (0.0419*P*)^2^] where *P* = (*F*_o_^2^ + 2*F*_c_^2^)/3
*wR*(*F*^2^) = 0.069	(Δ/σ)_max_ = 0.001
*S* = 0.98	Δρ_max_ = 0.18 e Å^−^^3^
2737 reflections	Δρ_min_ = −0.17 e Å^−^^3^
150 parameters	Extinction correction: *SHELXL97* (Sheldrick, 2008), Fc^*^=kFc[1+0.001xFc^2^λ^3^/sin(2θ)]^-1/4^
0 restraints	Extinction coefficient: 0.021 (2)
Primary atom site location: structure-invariant direct methods	Absolute structure: Flack (1983), 1128 Friedel pairs
Secondary atom site location: difference Fourier map	Flack parameter: 0.03 (5)

### Special details

**Table:**

Geometry. All e.s.d.'s (except the e.s.d. in the dihedral angle between two l.s. planes) are estimated using the full covariance matrix. The cell e.s.d.'s are taken into account individually in the estimation of e.s.d.'s in distances, angles and torsion angles; correlations between e.s.d.'s in cell parameters are only used when they are defined by crystal symmetry. An approximate (isotropic) treatment of cell e.s.d.'s is used for estimating e.s.d.'s involving l.s. planes.
Refinement. Refinement of *F*^2^ against ALL reflections. The weighted *R*-factor *wR* and goodness of fit *S* are based on *F*^2^, conventional *R*-factors *R* are based on *F*, with *F* set to zero for negative *F*^2^. The threshold expression of *F*^2^ > σ(*F*^2^) is used only for calculating *R*-factors(gt) *etc*. and is not relevant to the choice of reflections for refinement. *R*-factors based on *F*^2^ are statistically about twice as large as those based on *F*, and *R*- factors based on ALL data will be even larger.

### Fractional atomic coordinates and isotropic or equivalent isotropic displacement parameters (Å^2^)

**Table:**

	*x*	*y*	*z*	*U*_iso_*/*U*_eq_	
Cl1	0.70767 (6)	1.17100 (4)	0.53982 (4)	0.04673 (13)	
C1	0.54914 (14)	0.73267 (12)	0.53979 (11)	0.0203 (3)	
O1	0.43572 (10)	0.67397 (9)	0.56950 (7)	0.0245 (2)	
N1	0.63351 (13)	0.70808 (10)	0.45221 (9)	0.0233 (2)	
H1	0.720 (2)	0.7470 (16)	0.4421 (13)	0.028 (4)*	
C11	0.59541 (13)	0.84022 (13)	0.60027 (10)	0.0208 (3)	
C12	0.63710 (15)	0.94177 (12)	0.54747 (11)	0.0233 (3)	
H12	0.6474	0.9425	0.4720	0.028*	
C13	0.66355 (15)	1.04213 (13)	0.60606 (11)	0.0260 (3)	
C14	0.65300 (16)	1.04247 (14)	0.71660 (12)	0.0289 (3)	
H14	0.6727	1.1117	0.7558	0.035*	
C15	0.61351 (17)	0.94075 (15)	0.76869 (12)	0.0300 (3)	
H15	0.6069	0.9399	0.8444	0.036*	
C16	0.58335 (14)	0.83946 (14)	0.71154 (11)	0.0253 (3)	
H16	0.5547	0.7701	0.7479	0.030*	
C21	0.59346 (14)	0.61160 (13)	0.38088 (11)	0.0218 (3)	
H21	0.4778	0.5966	0.3868	0.026*	
C22	0.62972 (17)	0.64688 (13)	0.26615 (11)	0.0274 (3)	
H22A	0.7431	0.6654	0.2594	0.033*	
H22B	0.5690	0.7176	0.2476	0.033*	
C23	0.58727 (17)	0.54859 (16)	0.18917 (12)	0.0334 (4)	
H23A	0.4722	0.5353	0.1911	0.040*	
H23B	0.6161	0.5717	0.1156	0.040*	
C24	0.67181 (18)	0.43660 (15)	0.21786 (13)	0.0340 (3)	
H24A	0.7865	0.4471	0.2080	0.041*	
H24B	0.6367	0.3735	0.1696	0.041*	
C25	0.6385 (2)	0.40235 (15)	0.33291 (14)	0.0373 (4)	
H25A	0.7005	0.3322	0.3511	0.045*	
H25B	0.5255	0.3827	0.3405	0.045*	
C26	0.68006 (17)	0.50066 (13)	0.41084 (12)	0.0292 (3)	
H26A	0.6508	0.4774	0.4843	0.035*	
H26B	0.7950	0.5148	0.4092	0.035*	

### Atomic displacement parameters (Å^2^)

**Table:**

	*U*^11^	*U*^22^	*U*^33^	*U*^12^	*U*^13^	*U*^23^
Cl1	0.0806 (3)	0.02269 (18)	0.0369 (2)	−0.00841 (19)	−0.0039 (2)	0.00076 (18)
C1	0.0225 (5)	0.0208 (7)	0.0176 (5)	0.0040 (5)	−0.0026 (5)	0.0014 (6)
O1	0.0244 (4)	0.0256 (5)	0.0236 (5)	−0.0026 (4)	0.0023 (3)	0.0014 (4)
N1	0.0236 (5)	0.0234 (6)	0.0227 (5)	−0.0043 (4)	0.0028 (4)	−0.0057 (5)
C11	0.0203 (5)	0.0220 (7)	0.0202 (6)	0.0036 (5)	−0.0005 (4)	−0.0031 (6)
C12	0.0267 (5)	0.0245 (7)	0.0188 (6)	0.0028 (5)	−0.0008 (5)	−0.0020 (6)
C13	0.0304 (6)	0.0213 (7)	0.0261 (7)	0.0022 (5)	−0.0023 (5)	−0.0004 (6)
C14	0.0312 (6)	0.0291 (8)	0.0264 (7)	0.0030 (6)	−0.0031 (5)	−0.0095 (6)
C15	0.0318 (7)	0.0391 (9)	0.0190 (6)	0.0012 (6)	−0.0012 (5)	−0.0058 (6)
C16	0.0273 (6)	0.0292 (8)	0.0195 (6)	0.0012 (5)	0.0002 (5)	0.0004 (6)
C21	0.0231 (6)	0.0224 (7)	0.0199 (6)	−0.0027 (5)	−0.0008 (5)	−0.0054 (5)
C22	0.0355 (7)	0.0253 (7)	0.0214 (6)	0.0044 (6)	−0.0006 (5)	0.0001 (6)
C23	0.0360 (7)	0.0433 (10)	0.0208 (7)	0.0010 (7)	−0.0008 (5)	−0.0076 (7)
C24	0.0391 (8)	0.0306 (8)	0.0322 (7)	−0.0025 (6)	0.0058 (6)	−0.0122 (7)
C25	0.0532 (9)	0.0209 (8)	0.0377 (9)	−0.0028 (7)	0.0057 (7)	−0.0048 (7)
C26	0.0393 (7)	0.0229 (8)	0.0254 (7)	−0.0008 (6)	−0.0016 (6)	0.0018 (6)

### Geometric parameters (Å, °)

**Table:**

Cl1—C13	1.7383 (16)	C21—C22	1.5253 (19)
C1—O1	1.2337 (16)	C21—H21	1.0000
C1—N1	1.3410 (17)	C22—C23	1.528 (2)
C1—C11	1.5020 (18)	C22—H22A	0.9900
N1—C21	1.4641 (17)	C22—H22B	0.9900
N1—H1	0.868 (18)	C23—C24	1.517 (3)
C11—C12	1.387 (2)	C23—H23A	0.9900
C11—C16	1.3982 (18)	C23—H23B	0.9900
C12—C13	1.385 (2)	C24—C25	1.521 (2)
C12—H12	0.9500	C24—H24A	0.9900
C13—C14	1.388 (2)	C24—H24B	0.9900
C14—C15	1.380 (2)	C25—C26	1.534 (2)
C14—H14	0.9500	C25—H25A	0.9900
C15—C16	1.390 (2)	C25—H25B	0.9900
C15—H15	0.9500	C26—H26A	0.9900
C16—H16	0.9500	C26—H26B	0.9900
C21—C26	1.519 (2)		
			
O1—C1—N1	123.32 (13)	C21—C22—C23	110.54 (13)
O1—C1—C11	120.12 (11)	C21—C22—H22A	109.5
N1—C1—C11	116.52 (11)	C23—C22—H22A	109.5
C1—N1—C21	122.34 (11)	C21—C22—H22B	109.5
C1—N1—H1	117.6 (11)	C23—C22—H22B	109.5
C21—N1—H1	119.8 (11)	H22A—C22—H22B	108.1
C12—C11—C16	119.98 (13)	C24—C23—C22	111.43 (12)
C12—C11—C1	121.20 (11)	C24—C23—H23A	109.3
C16—C11—C1	118.62 (13)	C22—C23—H23A	109.3
C13—C12—C11	119.25 (12)	C24—C23—H23B	109.3
C13—C12—H12	120.4	C22—C23—H23B	109.3
C11—C12—H12	120.4	H23A—C23—H23B	108.0
C12—C13—C14	121.38 (14)	C23—C24—C25	110.85 (13)
C12—C13—Cl1	119.39 (11)	C23—C24—H24A	109.5
C14—C13—Cl1	119.22 (12)	C25—C24—H24A	109.5
C15—C14—C13	119.04 (14)	C23—C24—H24B	109.5
C15—C14—H14	120.5	C25—C24—H24B	109.5
C13—C14—H14	120.5	H24A—C24—H24B	108.1
C14—C15—C16	120.66 (13)	C24—C25—C26	111.71 (13)
C14—C15—H15	119.7	C24—C25—H25A	109.3
C16—C15—H15	119.7	C26—C25—H25A	109.3
C15—C16—C11	119.67 (15)	C24—C25—H25B	109.3
C15—C16—H16	120.2	C26—C25—H25B	109.3
C11—C16—H16	120.2	H25A—C25—H25B	107.9
N1—C21—C26	111.83 (11)	C21—C26—C25	110.41 (13)
N1—C21—C22	109.11 (11)	C21—C26—H26A	109.6
C26—C21—C22	110.99 (11)	C25—C26—H26A	109.6
N1—C21—H21	108.3	C21—C26—H26B	109.6
C26—C21—H21	108.3	C25—C26—H26B	109.6
C22—C21—H21	108.3	H26A—C26—H26B	108.1
			
O1—C1—N1—C21	2.9 (2)	C14—C15—C16—C11	0.9 (2)
C11—C1—N1—C21	−174.88 (11)	C12—C11—C16—C15	−0.08 (19)
O1—C1—C11—C12	−137.24 (12)	C1—C11—C16—C15	−174.93 (12)
N1—C1—C11—C12	40.62 (17)	C1—N1—C21—C26	−92.52 (14)
O1—C1—C11—C16	37.55 (17)	C1—N1—C21—C22	144.31 (12)
N1—C1—C11—C16	−144.60 (12)	N1—C21—C22—C23	−179.27 (11)
C16—C11—C12—C13	−1.17 (19)	C26—C21—C22—C23	57.06 (15)
C1—C11—C12—C13	173.54 (11)	C21—C22—C23—C24	−56.47 (16)
C11—C12—C13—C14	1.6 (2)	C22—C23—C24—C25	55.47 (17)
C11—C12—C13—Cl1	−177.30 (9)	C23—C24—C25—C26	−55.16 (18)
C12—C13—C14—C15	−0.8 (2)	N1—C21—C26—C25	−178.62 (12)
Cl1—C13—C14—C15	178.16 (10)	C22—C21—C26—C25	−56.52 (15)
C13—C14—C15—C16	−0.5 (2)	C24—C25—C26—C21	55.74 (17)

### Hydrogen-bond geometry (Å, °)

**Table:**

*D*—H···*A*	*D*—H	H···*A*	*D*···*A*	*D*—H···*A*
N1—H1···O1^i^	0.868 (18)	2.052 (18)	2.9161 (15)	173.3 (16)

Symmetry codes: (i) *x*+1/2, −*y*+3/2, −*z*+1.
